# The effects of scaling on age, sex and size relationships in Red-legged Partridges

**DOI:** 10.1038/s41598-018-20576-x

**Published:** 2018-02-01

**Authors:** Jesús Nadal, Carolina Ponz, Antoni Margalida

**Affiliations:** 1 Department of Animal Science, Division of Wildlife, Faculty of Life Sciences and Engineering, University of Lleida, Lleida, Spain; 2 Division of Conservation Biology, Institute of Ecology and Evolution, University of Bern, Bern, Switzerland

## Abstract

Wild birds differ in size according to their age and sex, adult birds being larger than juveniles. In the galliforms, males are larger than females, in contrast to some groups, such as the raptors, in which the females are larger. Size generally influences the rank hierarchy within a group of birds, although the age, sex, temperament and behaviour of an individual may override its size related rank order. The scaled size of birds according to age and sex affects their physiology and behaviour. Precise details of body-size differences by age and sex are poorly known in most partridge species. We measured 13,814 wild partridges in a homogenous population over 14 years of study to evaluate size differences within a uniform habitat and population management regime. We show that wild Red-legged Partridges have scaled mass, and body- and wing-lengths consistent with age/sex classes. Power functions between mass and body-length (as a proxy for walking efficiency), and between mass and wing-length (for flight efficiency) differ between juvenile females and males, and adult females and males. We discuss these findings and their physiological, behavioural and ecological implications.

## Introduction

A whole range of factors act to affect the size of any individual: its age and sex^1–4^, modified by its underlying genetics; its available food resources^5^; and its temperament (i.e., personality), which may affect growth through its behavioural characteristics^6–8^. In addition, habitat factors such as weather, availability of cover, and net primary production (NPP) of food resources may also affect breeding, growth and size^9,10^, and interactions with conspecific and heterospecific neighbours may affect growth and rate of maturation^11–13^. Therefore, growth rate, rate of maturation, and thus the ultimate size of an individual integrate the effects of a wide variety of ecological influences^14–17^, while climatic effects and density-dependent factors acting on population dynamics may also play a role^18^.

The social status of an individual bird living in a group depends upon its temperament (i.e., proactive/reactive, or aggressive/docile) and its position in the group’s various rank hierarchies (of size, age, sex, boldness), so that certain individuals within a group become dominant^19^. All of the birds in a flock contribute to its safeguarding and resource provisioning during daily activities that include feeding, vigilance, resting, preening, calling, and bathing^20,21^. The effectiveness of a group’s antipredator response will depend upon its cohesion and the effective participation of all of its members (i.e. the greater the number of individuals on high alert, the greater will be the distance at which danger is detected). The size of a bird therefore affects its social relationships, physiology and ecology in a variety of ways^22^.

The Red-legged Partridge (*Alectoris rufa*) (hereafter partridge) is a ground-dwelling species occurring in the wild in Mediterranean habitats and is sexually size-dimorphic (males being larger than females). It is a key prey and a small-game species^23^ that employs a social strategy (flocking) to maximise its foraging efficiency and to defend itself against predators. Rank hierarchies combine with age and sex related size differences to produce a social structure in partridge groups, although the success of the group is also subject to habitat conditions^24^.

In this paper we examine the effects of mass, body-length (bill tip to tip of tail), and wing-length of partridges to determine whether these traits differ among age/sex classes, since they may be important regulators of individual competition and cohesion within a flock^25^. We assumed that size would exert an influence on an individual’s behaviour^26^. If average size differs between age/sex classes, these differences could have implications for population fitness and the behavioural ecology of social groups^27,28^. To test this, we analysed the relationship between mass and body-length, wing-length, and age/sex class, by comparing isometric and allometric functions between mass-body-length and mass-wing-length^28^ for each age/sex class. The mass-body-length can be considered to be an index of walk-run fitness, and mass-wing-length as an index of flight fitness.

Our objectives were to: (i) determine whether different partridge age/sex classes differ in their body measurements; (ii) examine any scaled size relationships among age/sex classes; and (iii) investigate any power relationships between mass and body-length, and between mass and wing-length for age/sex classes.

## Results

**Figure 1:**
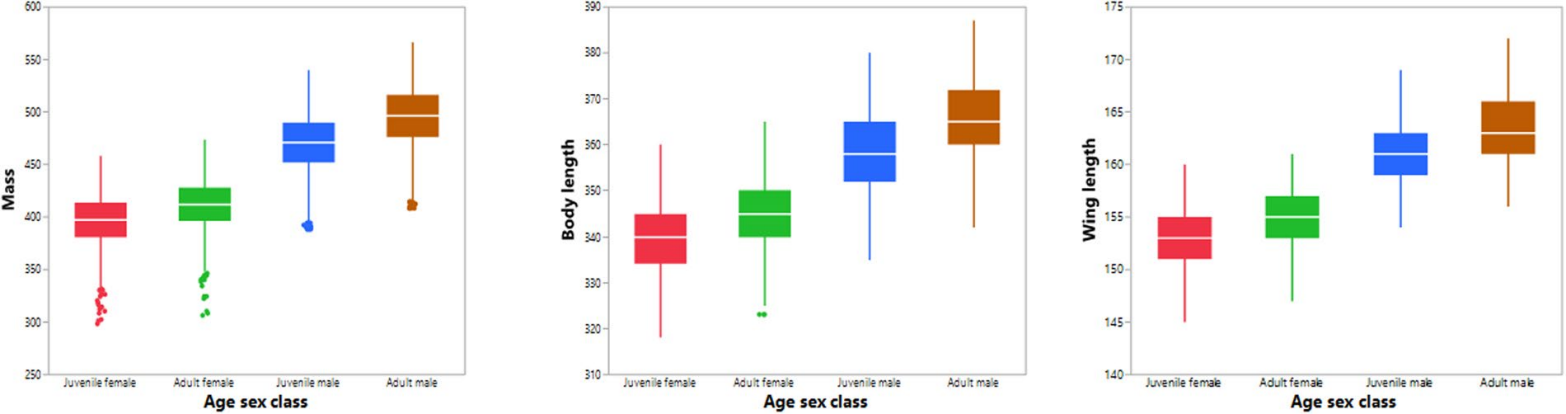
Scaled differences among the biometric measures studied (mass (g); body length and wing length, (mm)) between age/sex classes (juvenile female, adult female, juvenile male, adult male) in the Red-legged Partridge.

**Figure 2:**
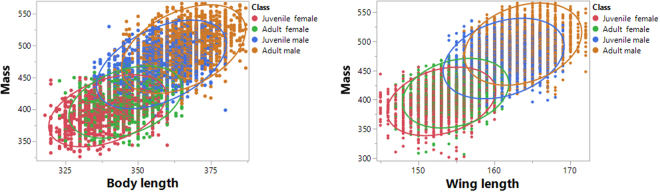
Associations between: mass (g) and body length (mm); and mass and wing length (mm) of Red-legged Partridges in Spain according to age and sex class. Ellipses include 95% of classes.

**Figure 3:**
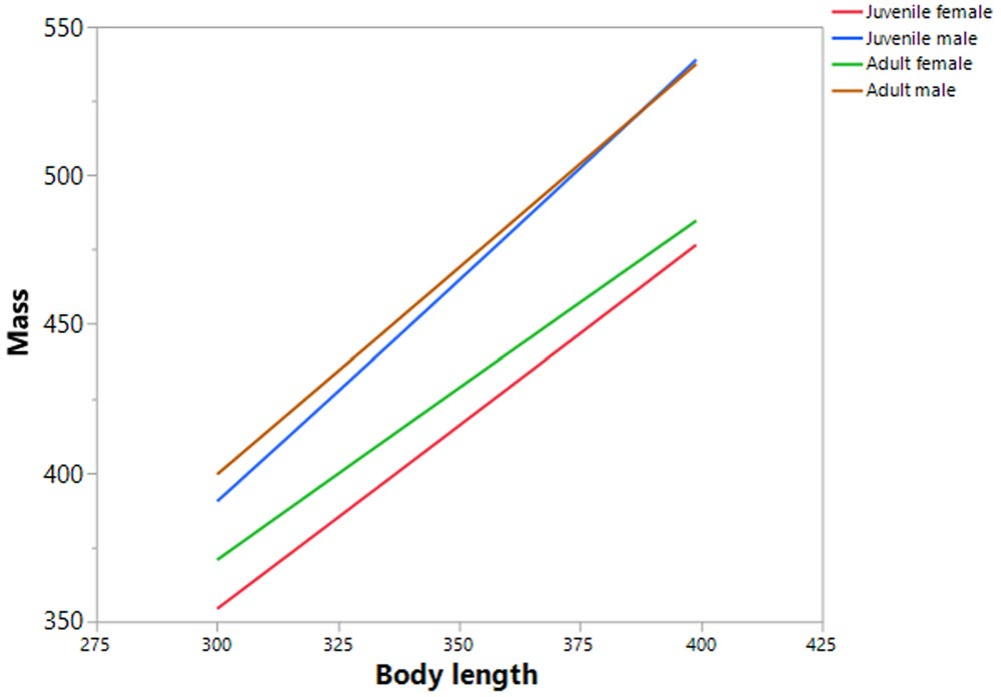
Power relation between mass (g) and body length (mm) for age/sex classes in the Red-legged Partridge.

**Figure 4:**
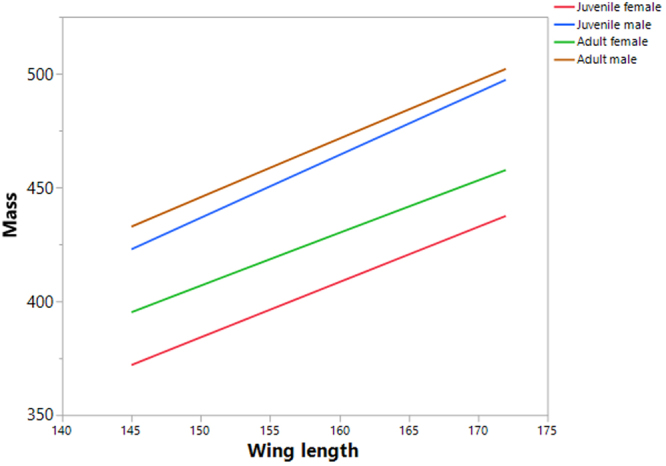
Power relation between mass (g) and wing length (mm) for age/sex classes in the Red-legged Partridge.

We found clear and significant differences in mass, body-length, and wing-length between the age/sex classes (Fig. 1, Table 1). 77.3% of the variability in mass was explained by body-length, wing-length, and age/sex class and their interactions. All of the regression effects were significant (Table 2). The relationships between mass and body-length, and mass and wing-length, were best described by a power function, rather than a linear one (Table 3). All of the parameters measured were scaled between age/sex classes in the following order: juvenile female < adult female < juvenile male < adult male. Overall, allometric relationships were found between mass and body-length (y = 0.0008X^2.27^) and mass and wing-length (y = 0.001X^2.54^) (Fig. 2). These equations showed allometric proportionality between the mass and body part variables. Moreover the power function changed according to age/sex class for mass with both body-length and wing-length (Figs 3 and 4, Table 4). In both allometric relationships, differences due to sex were greater than those for age, showing that sexual size dimorphism exerts a greater effect on mass than does rate of maturation. Indeed, as birds grow older the effect of sexual size dimorphism on mass increases while the effect of maturity decreases.

**Table 1:** Differences in body part measurements among age/sex classes (juvenile female, adult female, juvenile male, adult male) in Red-legged Partridges according to the analysis of variance (ANOVA).

	N	F	P
Mass	9,938	6,828.3	<0.0001
Total length	7,529	3,248.7	<0.0001
Wing length	11,539	7,155.5	<0.0001

**Table 2:** The effect of body length and wing length on mass in the Red-legged Partridge explained using a multiple regression model.

		F	P
Model		1,437.4	<0.0001
Effects	Age/sex classes	283.4	<0.0001
	Body length	395.8	<0.0001
	Wing length	124.6	<0.0001
	Class × Body length	4.5	0.004
	Class × Wing length	2.9	0.035
	Body length × Wing length	4.6	0.031
	Class x Body length × Wing length	2.6	0.048

**Table 3:** Corrected Akaike information criterion for linear and power functions in the relationships between mass and total length and wing length in the Red-legged Partridge.

	AICc lineal function	AICc power function
Mass respect total length	69,816	−18,960
Mass respect wing length	81,797	−21,196

**Table 4:** Power functions for mass in each age/sex class with respect to body length and wing length in the Red-legged Partridge, showing the results of lack of fit tests. Statistically significant results appear in bold type.

	Mass respect body length			Mass respect wing length		
		F	P		F	P
Juvenile female	0.94X^1.04^	1.54	**0.02**	3.29X^0.95^	1.54	0.09
Juvenile male	0.62X^1.13^	0.85	0.73	3.74X^0.95^	1.01	0.44
Adult female	1.71X^0.94^	0.94	0.58	5.47X^0.86^	1.82	**0.04**
Adult male	1.06X^1.04^	0.96	0.54	5.70X^0.87^	1.79	**0.03**

## Discussion

Our findings indicate that different age/sex classes of Red-legged Partridges can be distinguished by mass, body-length, and wing-length. Previous research on grouse^29^, and more recently on doves^30^, has shown the value of describing changes in mass during the annual cycle by age/sex classes, and then linking them to behavioural observations^31^ and moult^32^. Our data show that size differences between age/sex classes in partridges are influenced more by sex than by age, the differences between sexes being greater than those between different age classes^2^. Age is frequently associated with reproductive effort, moult, and migration^17,33,34^, while sex is associated with dimorphism, sexual selection, and parental division of reproductive effort^16,35,36^.

Our results show that partridge body-size is scaled between age/sex classes^37,38^. We can therefore hypothesize that body-size in each age/sex class might influence the social roles and position of each class in the flock hierarchy^39^. Differences in behaviour according to age/sex class may affect ecological processes such as selection, plasticity, heritability, survival, and population dynamics^9,40–44^. Several studies have shown that the scaling of body-size to age/sex class appears to enhance cohesion, efficiency in finding food, and anti-predator behaviour within a social group^1,6,21,45^. Further support for this suggestion comes from the observed allometric functions related to mobility: between mass and body-length (walking efficiency)^46^, and mass and wing-length (flight efficiency)^25^. Scaled size therefore appears to be a consequence of growth in early life; the transition from juvenile to adult and the appearance of sexual dimorphism^47–49^. Juveniles reach adult size and begin to mature three months after hatching, in about September. The rate and extent of early growth is one of the most important factors determining adult size^2^. Maturation begins once adult size is reached, and is complete by 12 months of age, after the completion of moult into adult plumage^49,50^. Previous research has focused on relating differences in animal size to geographical distribution, NPP, and global warming, because habitat factors exert a strong influence on animal size^51–54^. Our study was performed in a homogeneous habitat with seasonal changes in NPP, so that there is no influence of habitat on our data. In addition, our study population is homogeneous and increasing, with a high density, stable sex structure, and oscillating age structure^23^.

The structure of our data on size according to age and sex class is a logical grouping based on a probabilistic norm that takes account of the time at which a bird reaches maturity^55^. We assumed that the energetic requirements of a growing animal are determined by the relationship between its rate of heat production and its body-size^56^. Our samples, collected over 14 years in autumn, included juveniles and adults over three and 12 months of age respectively. The wider literature on harvesting shows that selection by age and sex within a catch is common in fish and large herbivores, and has evolutionary implications for the harvested populations^57,58^. This is not the case in wild partridges, which are shot at random after being driving through their habitat.

The social status of individual partridges within a flock depends on their individual size, maturity, and sex, and can influence the overall activities of the flock and thus its prospects of starvation or survival^10,24^. Our findings support the idea that individual size (mass, body-length, and wing-length) may influence individual physiology and behaviour, and that partridge groups may take advantage of the scaled size in age/sex classes to improve the population’s ability to survive and reproduce. Behavioural studies should be conducted to further examine these relationships.

## Material and Methods

### Ethics statement

The study was conducted in full compliance with Spanish laws and regulations, including a license from “Las Ensanchas” to sample shot partridges. The protocol was approved by the Committee on the Ethics of Animal Experiments of the University of Lleida (Ref.1998–2012/05).

### Data collection

We examined wild partridges shot at Las Ensanchas, a small-game hunting estate in the Jabalón River Basin in Ciudad Real, Spain (38°39’ N, 3°13′ W, 790–840 m a.s.l.). This area comprises a mix of open woodland with pastures and cultivated land containing a mosaic of cereal crops, fallow, natural pastures and scrubland with scattered holm oaks (*Quercus ilex*). Overall, 75% of the estate is covered with herbaceous vegetation and 25% with shrubland. We studied still-warm, recently shot wild partridges between 1998 and 2011, as a representative sample of the whole population. Age was determined by examining primary feathers, and sex by spur characteristics. We weighed birds in the field using a digital balance with a 1 g scale. Body length was measured^23^ from beak to tail-tip, with the body flattened against the ruler. One wing was taken from all birds (cut through the ulna-radius) and prepared for study in the laboratory.

We combed the wings and washed them (if necessary), after which they were oven dried for 15 days at 40 °C. We recorded wing length to the nearest 0.5 mm (using standard practice, from wrist to wingtip with the folded wing stretched and flattened against the ruler), and repeated the measurement to confirm the recorded values. The precision of our measurements depended upon the skill of the recorder, which increased with experience^59^. One of the authors (JN) performed all of the field measurements and the other (CP) all of the laboratory measurements. Over the 14 years of the study, we examined 13,814 wild partridges, 77% captured in October, 20% in November and 3% in December. Not all of the birds examined were used for all of the measurements since some were mutilated, lacked key body-parts, or had broken or moulting feathers, and these individuals were eliminated from the data analysed.

The onset of sexual maturity is affected by age and size^55^. This appears to be the norm in our study and we assumed that maturation is a deterministic process. We classified birds by primary feather and spur characteristics as either juvenile female, adult female, juvenile male, or adult male. Of the birds examined: 9,938 (72%) partridges provided usable mass (weight) measurements; 7,529 (54%) gave body length; and 11,539 (83.5%) gave wing length.

### Statistical analyses

We verified data normality using the Shapiro-Wilk test, and used analysis of variance (ANOVA) to test size differences between all age/sex classes^60^, and to build graphs to show the scaling of size in age/sex classes. We used multiple regressions to examine interactions between size and body length, wing length and class. Although the large sample size overdrives the influence of multicollinearity on interpretation of the regression coefficients, we calculated variance inflation factor (VIF) to test for multicollinearity, and to verify the residual distribution and autocorrelation. We pooled age/sex classes to examine possible relationships between mass and total length, and mass and wing length, using linear and power functions. We applied the corrected Akaike information criterion (AICc) to select between linear and power models^28,61,62^. In each age/sex class we used power equations to examine allometric relationships between mass and total length, and mass and wing length^63^, and used a lack of fit test to check if the resulting models could be improved.

### Availability of data and materials

The datasets analysed during the current study are available from the corresponding author on reasonable request.
